# Multimodal MRI to elucidate cross-organ coupling mechanisms of the heart-brain axis

**DOI:** 10.3389/fcvm.2026.1914265

**Published:** 2026-09-28

**Authors:** Wanqing Tan, Xinling Han, Xuliang Wang, Mingsong Tang, Shilan Li, Hong Liu, Jing Zhang, Jianfei Wang, Guangyao Liu

**Affiliations:** 1 Department of Magnetic Resonance Imaging, Lanzhou University Second Hospital, Lanzhou, China; 2 Department of Cardiology, Lanzhou University Second Hospital, Lanzhou, China

**Keywords:** Alzheimer's disease, heart failure, heart-brain axis, magnetic resonance imaging, stroke

## Abstract

Cardiovascular and cerebrovascular diseases (CCVDs) are leading causes of mortality and disability worldwide, and their development is closely linked to bidirectional interactions between the heart and the brain. The heart-brain axis, a systemic framework that integrates neural, humoral, and inflammatory pathways, provides an important theoretical basis for understanding heart-brain comorbidities. With continuous advances in magnetic resonance imaging(MRI), research in this field has progressively shifted from the traditional single-organ diagnostic model to cross-organ and system-oriented strategies for prevention and management. Centered on cardiac magnetic resonance (CMR) and brain MRI, and complemented by quantitative, diffusion, functional, ultra-high-field, and metabolic imaging techniques, multimodal MRI has become a powerful tool for comprehensively characterizing myocardial remodeling, alterations in brain networks, and their coupling relationships. These techniques have been widely applied in studies of representative heart-brain comorbidities, including cardiac dysfunction associated with acute brain injury, cognitive impairment related to heart failure, and cardiovascular abnormalities associated with neurodegenerative diseases, such as Alzheimer's disease (AD). Such studies have not only revealed structural and functional alterations in the heart and brain but have also provided important insights into potential mechanisms, including autonomic imbalance, neuroendocrine activation, and inflammatory responses. However, current heart-brain axis research remains largely based on correlational analyses, whereas causal inference and characterization of dynamic mechanisms remain insufficient. In addition, significant challenges persist in multimodal data integration and clinical translation. Overall, MRI is driving heart-brain axis research from structural characterization toward mechanistic investigation. In the future, integrating imaging, physiological, and genetic information may enable early identification, risk stratification, and precision intervention for heart-brain comorbidities, thereby promoting a transition in medical practice from single-organ management to systemic prevention and treatment.

## 1 Introduction

CCVDs are among the leading causes of mortality and disability worldwide, and their disease burden continues to rise, posing a major global public health challenge (1, 2). Conventional diagnostic and therapeutic strategies are largely organ-centered, focusing on the independent evaluation and management of cardiac or cerebral disorders (3). However, such approaches often overlook the complex and dynamic pathophysiological interactions between the heart and the brain, thereby limiting a comprehensive understanding of disease mechanisms and hindering the development of integrated prevention and treatment strategies. In recent years, the heart-brain axis has emerged as an important conceptual framework for understanding interactions between the cardiovascular and central nervous systems. This framework emphasizes a bidirectional communication network mediated by autonomic regulation, humoral signaling, inflammatory and immune pathways, and neurovascular coupling (3, 4). Through these mechanisms, the heart-brain axis plays a central role in maintaining systemic homeostasis and contributes to the initiation and progression of multiple heart-brain comorbidities. Accordingly, investigating heart-brain interactions from a systemic perspective is essential for elucidating the pathological basis of these disorders and facilitating a transition from conventional single-organ medicine toward systems-based approaches.

Advances in MRI have provided new opportunities for heart-brain axis research, particularly through the development of multimodal MRI techniques (5). MRI enables high-resolution assessment of cardiac structure, function, and tissue characteristics, while complementary neuroimaging modalities—including functional magnetic resonance imaging (fMRI), diffusion tensor imaging (DTI), arterial spin labeling (ASL), and magnetic resonance spectroscopy (MRS)—allow characterization of cerebral abnormalities at structural, functional, microstructural, and metabolic levels (6). Together, these techniques provide cross-organ and multiscale imaging evidence for understanding heart-brain interactions. Moreover, MRI-based research has progressively evolved from single-organ evaluation to cross-organ, multimodal, and network-oriented integrative analyses, offering new perspectives on investigating dynamic heart-brain coupling and systemic pathological mechanisms (7).

Importantly, the heart-brain axis is not a single disease entity, but rather a complex cross-organ interaction network involved in a broad spectrum of pathological conditions, including acute brain injury, chronic cardiovascular diseases, and neurodegenerative disorders (8). These conditions may induce bidirectional heart-brain dysregulation and synergistic injury through mechanisms such as autonomic imbalance, impaired neurovascular coupling, hemodynamic alterations, inflammatory and immune activation, and metabolic dysfunction. For example, cardiac dysfunction associated with acute brain injury represents a typical brain-to-heart acute stress injury pattern. In contrast, brain injury and cognitive impairment secondary to heart failure reflect chronic heart-to-brain hypoperfusion and vascular remodeling. In addition, cardiovascular abnormalities associated with neurodegenerative diseases indicate that the heart-brain axis may represent a state of systemic network dysregulation. Collectively, these disorders constitute the major disease spectrum in current heart-brain axis research and provide important clinical scenarios for cross-organ, multiscale, and multimodal MRI investigations.

Accordingly, this review summarizes recent advances in the application of MRI to representative heart-brain axis disorders. Further, it discusses the potential value of multimodal MRI in elucidating cross-organ pathological mechanisms, systemic network remodeling, and the translation of precision medicine.

## 2 Physiological and pathological basis of the heart-brain axis

The physiological basis of the heart-brain axis primarily involves three interconnected pathways: neural, mechanical, and biochemical mechanisms (9). The neural pathway is centered on the autonomic nervous system and the central autonomic network (CAN), enabling rapid bidirectional communication between the brain and the cardiovascular system. The mechanical pathway depends on baroreceptors and mechanosensitive structures expressing ion channels such as Piezo, which transmit alterations in blood pressure and hemodynamics to the central nervous system through peripheral and cerebrovascular pathways. The biochemical pathway involves multiple endogenous mediators, including neurotransmitters, hormones, and cardiac-derived factors such as natriuretic peptides, which collectively regulate neural and cardiovascular function. These mechanisms have been systematically summarized in a review by Gaetano Valenza et al. (10). A key regulatory circuit within the heart-brain axis is the “baroreceptor-brainstem regulatory system,” which dynamically modulates heart rate and blood pressure on a second-to-second timescale to maintain cardiovascular homeostasis. Under pathological conditions such as stress or hypertension, this regulatory system may undergo set-point resetting. In parallel, neuroendocrine pathways, including the hypothalamic-pituitary-adrenal axis, interact with cardiac-derived secretory factors to mediate bidirectional regulation between the heart and the brain. In addition, cardiac pulsation itself may directly influence central neural activity through mechanosensitive channels. These multimodal signals are integrated within the CAN, which primarily includes the brainstem, hypothalamus, insular cortex, anterior cingulate cortex, and amygdala. Beyond regulating visceral homeostasis, the CAN also plays an important role in emotional and cognitive processing. Consequently, emotional and cognitive activities can rapidly modulate cardiac function through top-down pathways, whereas cardiac activity can influence perception, pain processing, and emotional experience through bottom-up signaling.

Overall, the relationship between the heart and the brain is characterized by highly coupled, bidirectional, and dynamic regulation that cannot be fully explained by a single causal mechanism. Its long-term functional state is shaped by both genetic and environmental factors and contributes to the initiation and progression of multiple psychosomatic disorders (11). At the pathological level, current research mainly focuses on cardiac dysfunction associated with acute brain injury, cognitive impairment related to heart failure, and cardiovascular abnormalities associated with neurodegenerative diseases. Despite their clinical heterogeneity, these conditions are linked by several shared mechanisms, including autonomic imbalance, neuroendocrine activation, inflammatory responses, and neurovascular dysfunction (12).

## 3 Multiscale magnetic resonance imaging for investigating heart-brain axis coupling mechanisms

Currently, multimodal MRI has become a key tool for investigating the heart-brain axis. Brain MRI encompasses a range of techniques, including structural MRI, fMRI, diffusion MRI, perfusion imaging, ASL, MRS, and contrast-enhanced MRI, which collectively enable comprehensive assessment of brain morphology, connectivity, perfusion, and metabolism. Similarly, CMR incorporates cine MRI, late gadolinium enhancement (LGE), T1/T2 mapping, perfusion-weighted imaging (PWI), and four-dimensional flow MRI (4D Flow MRI), providing detailed evaluation of cardiac structure, function, tissue characteristics, perfusion, and hemodynamics. By characterizing complementary aspects of cerebral and cardiac physiology, these imaging techniques together constitute the foundation of multimodal MRI approaches for investigating cross-organ coupling mechanisms within the heart-brain axis (Figure 1) (7).

**Figure 1:**
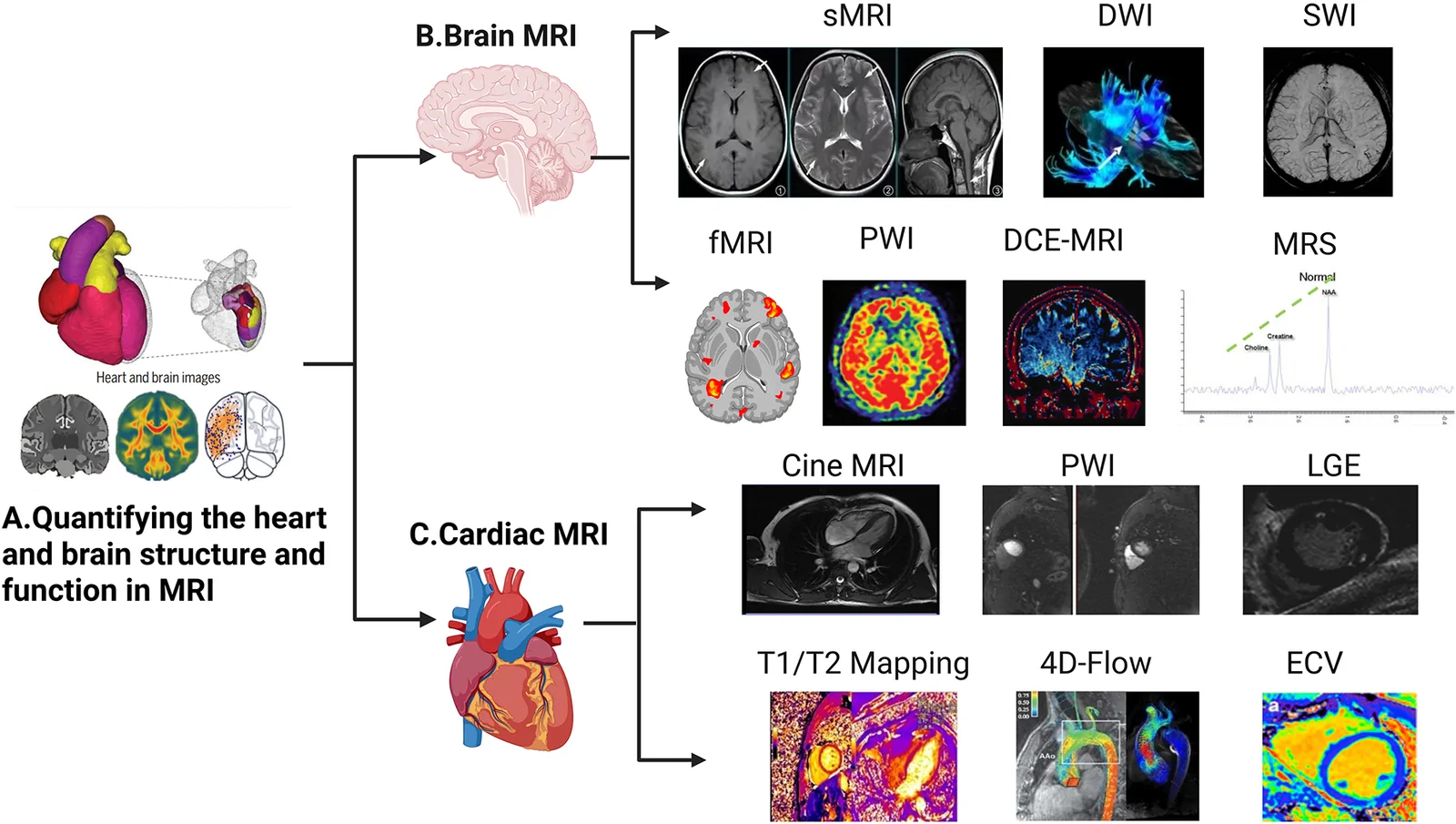
Overview of the MRI. **(A)** Quantifying the heart and brain structure and function in MRI. **(B)** Brain MRI. **(C)** Cardiac MRI.

### 3.1 Macroscopic structural remodeling: MRI phenotypes of coordinated heart-brain injury

Multimodal MRI can reveal coordinated remodeling of the heart-brain axis at the macroscopic structural level. CMR, currently regarded as the gold standard for assessing cardiac structure, function, and tissue characteristics, enables comprehensive evaluation of ventricular remodeling, myocardial fibrosis, edema, and perfusion abnormalities through techniques such as cine MRI, LGE, and T1/T2 mapping. Among these techniques, cine MRI enables accurate quantification of ventricular volumes, ejection fraction, and myocardial strain. LGE is widely used to identify focal fibrosis and scar formation, whereas T1/T2 mapping and extracellular volume fraction (ECV) measurements allow quantitative assessment of diffuse myocardial fibrosis and tissue remodeling (13). Correspondingly, structural brain MRI, including T1-weighted and T2/fluid-attenuated inversion recovery (FLAIR) imaging, can detect representative cerebral injury phenotypes such as brain atrophy, cerebral infarction, and white matter hyperintensities (WMHs) (14). An increasing number of studies have demonstrated significant associations between cardiac remodeling and structural brain abnormalities. For example, patients with heart failure frequently exhibit reduced gray matter volume (15) in regions including the hippocampus, prefrontal cortex, and cingulate cortex, whereas patients with atrial fibrillation (16) or cardiomyopathy frequently exhibit cerebral small vessel disease markers, including increased WMH burden and silent cerebral infarction and manifestations of cerebral small vessel disease (17). These findings suggest that cardiac and cerebral injuries are not isolated events (14) but rather components of a systemic remodeling process jointly driven by chronic hypoperfusion, vascular remodeling, and autonomic imbalance (18).

Therefore, structural MRI is valuable not only for identifying single-organ injury, but also for providing critical imaging evidence to characterize “structural co-remodeling” within the heart-brain axis. This approach is gradually shifting research on heart-brain comorbidities from the description of localized lesions toward cross-organ systemic phenotyping and integrative network-based analysis (19). Current evidence regarding the temporal sequence and causal direction of heart-brain remodeling remains limited. Most available studies are cross-sectional in nature and primarily demonstrate concurrent associations between cardiac abnormalities, such as reduced myocardial strain, ventricular remodeling, and myocardial fibrosis, and cerebral alterations, including white matter hyperintensities, hippocampal atrophy, and disrupted functional connectivity. However, longitudinal MRI studies capable of determining whether cardiac remodeling precedes cerebral injury, or conversely whether cerebral alterations contribute to subsequent cardiac dysfunction, remain scarce (20).

### 3.2 Diffusion MRI reveals microstructural network injury in the heart-brain axis

Beyond macroscopic structural alterations, diffusion MRI further reveals microstructural network injury within the heart-brain axis (21). Techniques such as diffusion-weighted imaging (DWI), DTI, and neurite orientation dispersion and density imaging (NODDI) enable multidimensional assessment of cerebral microstructural abnormalities, including white matter fiber integrity, axonal density, and neural network organization. In heart-brain axis research, these techniques are particularly valuable for evaluating the structural integrity of the CAN (22). The CAN primarily includes the insular cortex, anterior cingulate cortex, amygdala, thalamus, and brainstem, and is responsible for integrating emotional, cognitive, and visceral autonomic information (22). DTI studies have shown that patients with heart failure and neurodegenerative diseases commonly exhibit reduced white matter integrity within frontolimbic systems and brainstem autonomic pathways (23), characterized by decreased fractional anisotropy and increased mean diffusivity (24). These findings indicate disruption of autonomic regulatory networks, which may further contribute to sympathetic-parasympathetic imbalance and abnormal cardiovascular regulation. In addition, microstructural MRI provides important mechanistic insights into how progressive degeneration of brain networks may contribute to cardiovascular dysfunction. For example, in patients with Parkinson's disease and AD (25), abnormalities in white matter connectivity are associated not only with cognitive decline, but also with reduced heart rate variability and impaired blood pressure regulation (26). These findings suggest that degeneration of autonomic neural networks may represent a critical mechanistic link between neurodegenerative pathology and cardiovascular dysfunction.

Therefore, diffusion MRI is shifting heart-brain axis research from conventional anatomical observation toward the evaluation of “network integrity” (22) and provides important imaging evidence for understanding the role of the autonomic nervous system in cross-organ information transfer and systemic network dysregulation.

### 3.3 Functional network coupling and dynamic heart-brain regulation

fMRI further elucidates the dynamic regulatory mechanisms of the heart-brain axis from the perspective of functional connectivity. In particular, resting-state functional MRI (rs-fMRI) enables assessment of functional synchronization among distributed brain regions in the absence of task performance, thereby facilitating the identification of brain network abnormalities associated with autonomic regulation. Current evidence suggests that the default mode network (DMN), salience network, and limbic system constitute key neural substrates involved in heart-brain axis regulation (27). Among these networks, the insula-anterior cingulate cortex pathway is considered a critical hub for autonomic control, and its dysfunction is closely associated with sympathetic overactivation, reduced heart rate variability, and impaired emotional regulation. Functional connectivity abnormalities within these networks have been observed in patients with heart failure and psychiatric disorders (28), suggesting that distinct disease states may share common mechanisms of heart-brain coupling (29). Importantly, rs-fMRI has shifted heart-brain axis research from the analysis of focal lesions toward the investigation of network-level dysfunction (30). Increasing evidence indicates that cardiovascular abnormalities affect not only regional cerebral perfusion but also induce widespread reorganization of functional brain networks. For example, patients with chronic heart failure frequently exhibit reduced connectivity between the DMN and executive control network (28), whereas patients with anxiety and depression more commonly demonstrate salience network hyperactivation and abnormal limbic coupling (31). These findings suggest that functional brain network remodeling may represent a key neural basis underlying heart-brain comorbidities.

### 3.4 Cerebral perfusion abnormalities and neurovascular coupling dysfunction

Perfusion MRI provides an important tool for characterizing hemodynamic abnormalities within the heart-brain axis. ASL enables noninvasive quantification of cerebral blood flow, whereas dynamic contrast-enhanced MRI (DCE-MRI) allows further assessment of blood-brain barrier (BBB) integrity and microvascular permeability (32). Chronic cerebral hypoperfusion is considered a key mechanism linking cardiovascular disease and brain dysfunction (14). Studies have shown that patients with heart failure and atrial fibrillation often exhibit reduced cerebral perfusion globally, as well as in critical regions such as the hippocampus, and these reductions are associated with the severity of cognitive impairment (23). These findings suggest that persistent reductions in cardiac output, together with impaired cerebral autoregulation, may jointly contribute to structural and network-level brain injury. In addition, following stroke or acute brain injury, excessive sympathetic activation and inflammatory responses can lead to endothelial dysfunction and increased BBB permeability. DCE-MRI studies have demonstrated that BBB disruption not only contributes to acute neuroinflammatory responses but may also promote subsequent white matter injury and long-term cognitive decline (33). Furthermore, patients with neurodegenerative diseases also exhibit impaired neurovascular coupling (34), suggesting that dysregulation of cerebral blood flow represents a shared pathological mechanism across multiple heart-brain comorbid conditions.

Therefore, perfusion MRI not only enables the assessment of focal ischemia but also provides critical insight into dynamic blood flow-vascular-neural interactions within the heart-brain axis (34), offering an important framework for understanding chronic hypoperfusion and cerebrovascular dysfunction in heart-brain comorbidities.

### 3.5 Metabolic and molecular abnormalities: From structural imaging to biological mechanisms

With the development of metabolic MRI, heart-brain axis research has expanded beyond structural and functional characterization toward molecular and metabolic investigation of cross-organ interactions (35). Advanced techniques, including MRS multinuclear MRI (^31^P-MRS and ^23^Na MRI), and quantitative susceptibility mapping (QSM), enable *in vivo* assessment of key biological processes, such as energy metabolism, ionic homeostasis, oxidative stress, and metal deposition (36). Increasing clinical evidence suggests that these approaches can identify metabolic signatures associated with both cardiovascular and neurological disorders, thereby providing new insights into the biological mechanisms underlying heart-brain coupling.

Among these techniques, ^31^P-MRS has been extensively applied in patients with heart failure and ischemic cardiomyopathy, demonstrating reduced phosphocreatine-to-adenosine triphosphate (PCr/ATP) ratios, which indicate impaired myocardial energy metabolism and mitochondrial dysfunction (37). Importantly, these metabolic abnormalities may occur before overt structural remodeling, highlighting the potential value of ^31^P-MRS as an early biomarker of myocardial dysfunction (38). Similarly, ^23^Na MRI provides unique information regarding myocardial sodium homeostasis disturbances in ischemic and fibrotic tissues, offering a potential non-invasive approach for assessing myocardial ionic dysregulation associated with injury and remodeling.

Metabolic MRI has also provided important evidence of brain metabolic alterations associated with cardiovascular diseases. Brain MRS studies in patients with heart failure and cognitive impairment have demonstrated abnormal metabolite profiles, including reduced N-acetylaspartate levels and disrupted neurotransmitter-related metabolism, suggesting neuronal dysfunction secondary to cardiac impairment (39). Furthermore, QSM has revealed abnormal iron accumulation in neurodegenerative disorders such as AD and Parkinson's disease, with increased iron deposition associated with disease progression and cognitive decline (40, 41). These findings suggest that metabolic and molecular imaging may provide a valuable framework for investigating the interplay between vascular dysfunction, neurodegeneration, and heart-brain axis disorders.

Beyond MRI-based metabolic imaging, positron emission tomography (PET) provides complementary molecular information regarding glucose metabolism, inflammation, and protein deposition through disease-specific radiotracers (42). ^18F-fluorodeoxyglucose (FDG)-PET has demonstrated abnormal metabolic remodeling in cardiovascular diseases and characteristic cerebral metabolic patterns in neurodegenerative disorders such as Alzheimer's disease. Moreover, hybrid PET/MRI platforms enable simultaneous assessment of cardiac remodeling, cerebral metabolism, inflammation, and molecular alterations, offering a comprehensive approach for investigating the complex biological mechanisms of heart-brain interactions (43).

### 3.6 Integrated heart-brain imaging and cross-organ dynamic coupling

In recent years, MRI research has evolved from single-organ assessment toward integrated cross-organ imaging. One-stop heart-brain MRI enables simultaneous acquisition of cardiac and cerebral structural, functional, and perfusion information within a single examination, allowing systematic evaluation of dynamic interactions along the heart-brain axis. Studies combining (ECG), electroencephalography, and MRI have demonstrated temporal synchronization between brain activity and cardiac rhythms. By integrating brain functional connectivity, heart rate variability, and autonomic nervous system indices (27), it is possible to construct dynamic brain-heart coupling models that characterize directional information flow and temporal evolution across different disease states. Meanwhile, advances in graph theory analysis and network neuroscience have driven a shift in heart-brain axis research from localized abnormalities toward system-level network modeling. Metrics such as nodal centrality, modularity, and global efficiency enable quantitative assessment of information integration capacity and dynamic organizational properties between brain networks and the cardiovascular system.

In addition, developments in flexible coil technology, ultra-high-field MRI (44), and deep learning-based reconstruction algorithms have further improved spatial resolution and scanning efficiency, making integrated brain-heart imaging increasingly feasible. These advances may enable continuous, cross-organ, and multi-timescale dynamic observation, providing a new paradigm for elucidating directional propagation and system-level coupling mechanisms within the heart-brain axis.

## 4 Multimodal MRI reveals cross-organ coupling mechanisms in heart-brain axis disorders

Multimodal MRI has emerged as a powerful tool for investigating the heart-brain axis by providing comprehensive assessments of structural, functional, vascular, and metabolic alterations in both organs. Through the integration of cardiac and cerebral imaging biomarkers, it enables the evaluation of bidirectional heart-brain interactions and offers mechanistic insights into the processes underlying co-remodeling and co-atrophy. Figure 2 summarizes the major heart-brain interaction patterns and the corresponding multimodal MRI approaches used to characterize these changes. (Figure 2).

**Figure 2:**
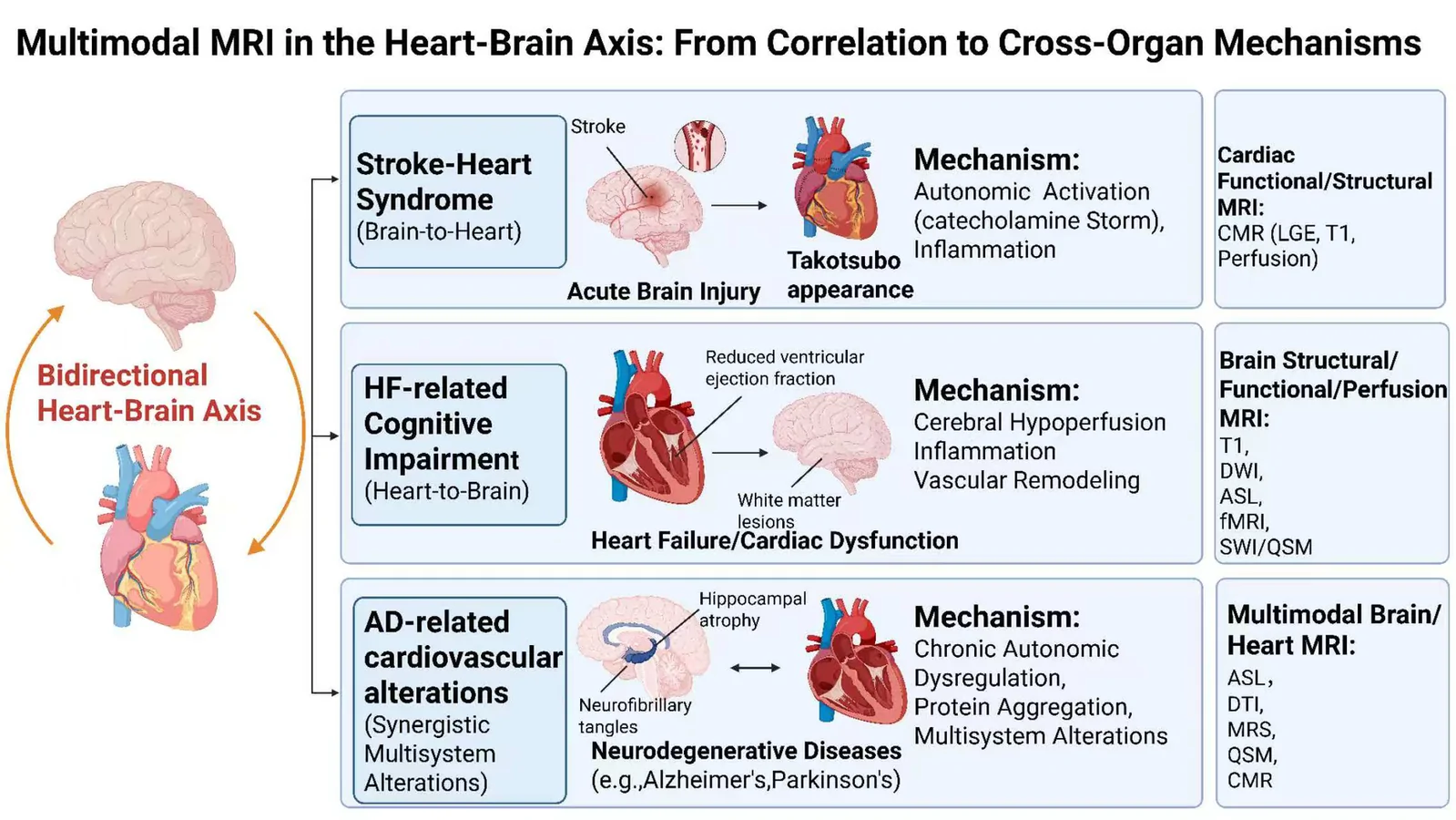
Multimodal MRI characterization of heart-brain axis remodeling. This figure illustrates three representative patterns of heart-brain interaction. (1) Stroke-heart syndrome, in which acute brain injury triggers myocardial dysfunction through autonomic activation, catecholamine excess, and inflammatory responses. (2) Heart failure-related cognitive impairment, where reduced cardiac output contributes to chronic cerebral hypoperfusion, vascular remodeling, and white matter injury. (3) Neurodegenerative disease-associated cardiovascular atrophy, characterized by persistent autonomic dysregulation and parallel degeneration of cardiac and cerebral structures.

Across representative heart-brain disorders, converging multimodal MRI evidence has revealed recurring cross-organ imaging signatures. Abnormal insular functional connectivity is frequently associated with impaired myocardial strain, whereas hippocampal atrophy and white matter injury commonly accompany myocardial fibrosis and adverse cardiac remodeling (39). These reproducible imaging phenotypes support the concept that heart and brain dysfunction evolve through interconnected autonomic, vascular, inflammatory, and metabolic pathways, highlighting the unique value of multimodal MRI for characterizing systemic heart-brain coupling.

### 4.1 Cardiac dysfunction associated with acute brain injury

Acute brain injuries such as ischemic stroke can rapidly induce significant cardiac dysfunction (45), representing a classic brain-to-heart pathological pattern. The underlying mechanisms are primarily related to injury of key autonomic regulatory centers, including the insular cortex, thalamus, and brainstem (46), which triggers a sympathetic storm and catecholamine-mediated cardiotoxicity (45). Acute brain injury also activates the hypothalamic-pituitary-adrenal axis (45), leading to excessive catecholamine release. This cascade contributes to neurogenic myocardial injury, myocardial stunning, and microcirculatory dysfunction. In addition, catecholamine surge can disrupt intracellular calcium homeostasis and impair mitochondrial function in cardiomyocytes, ultimately resulting in apical ballooning of the left ventricle, known as Takotsubo syndrome (TTS) (47). This neuro-cardiac interaction is characterized by a dynamic and bidirectional relationship. On one hand, excessive sympathetic activation may further aggravate cardiac injury through neurogenic myocardial edema and inflammatory responses. On the other hand, reduced cardiac output caused by myocardial stunning can exacerbate cerebral hypoperfusion, thereby worsening ischemic brain injury and creating a vicious cycle of injury propagation. Cardiac dysfunction following stroke represents one of the most representative models of acute neurogenic myocardial injury in heart-brain axis research and has recently been conceptualized as stroke-heart syndrome (48). Epidemiological studies have shown that patients with TTS have a significantly increased risk of stroke (49), while the incidence of TTS in patients with acute ischemic stroke is approximately 1.2% (47), suggesting a close bidirectional pathological relationship between the two conditions. In patients undergoing both cardiac and brain MRI, typical imaging features of TTS may coexist with stroke-related cerebral lesions, further supporting the presence of brain-heart coupling (50). The insular cortex, as a key autonomic regulatory hub, may trigger excessive sympathetic activation and myocardial injury when affected by ischemia (46). In addition, brainstem ischemia-related autonomic dysfunction is also considered an important mechanism underlying acute neurogenic cardiac injury. Conversely, cardiac disorders such as atrial fibrillation and heart failure can further increase the risk of stroke and cerebral hypoperfusion, forming a sustained heart-brain pathological cascade (51). MRI plays a crucial role in elucidating stroke-related cardiac injury. Brain MRI, particularly DWI, FLAIR, and perfusion imaging, enables precise delineation of ischemic core and penumbra (52). Lesions involving the insula, thalamus, and brainstem are closely associated with arrhythmias, myocardial injury, and hemodynamic instability, as these regions are directly involved in baroreflex regulation and autonomic output (52). Damage to these structures can rapidly disrupt the balance between sympathetic and parasympathetic activity. Notably, some ischemic stroke patients may present with DWI-negative findings (53), which are generally associated with relatively favorable clinical outcomes. On the cardiac side, CMR can identify characteristic features of TTS (54), including regional wall motion abnormalities, myocardial edema, and the absence of obstructive coronary artery disease. Techniques such as T1/T2 mapping and LGE further enable quantitative assessment of myocardial tissue alteration and microvascular injury. Overall, this brain injury-triggered cardiac response reflects, at its core, catecholamine-mediated neurogenic myocardial toxicity and acute microcirculatory dysfunction occurring on a short temporal scale (48).

Representative multimodal MRI findings associated with acute stroke-related cardiac injury are illustrated in Figure 3. Brain MRI demonstrates acute ischemic lesions involving key autonomic regulatory regions, particularly the insular cortex, with DWI, ADC, and FLAIR sequences confirming cerebral ischemic injury (55). Subsequent CMR imaging reveals characteristic manifestations of neurogenic myocardial injury, including left ventricular dysfunction, apical ballooning, and myocardial edema (56). Together, these multimodal imaging findings illustrate the disruption of central autonomic regulation and catecholamine-mediated myocardial injury that underlie stroke-heart syndrome, providing imaging evidence of bidirectional brain-heart interactions.

**Figure 3:**
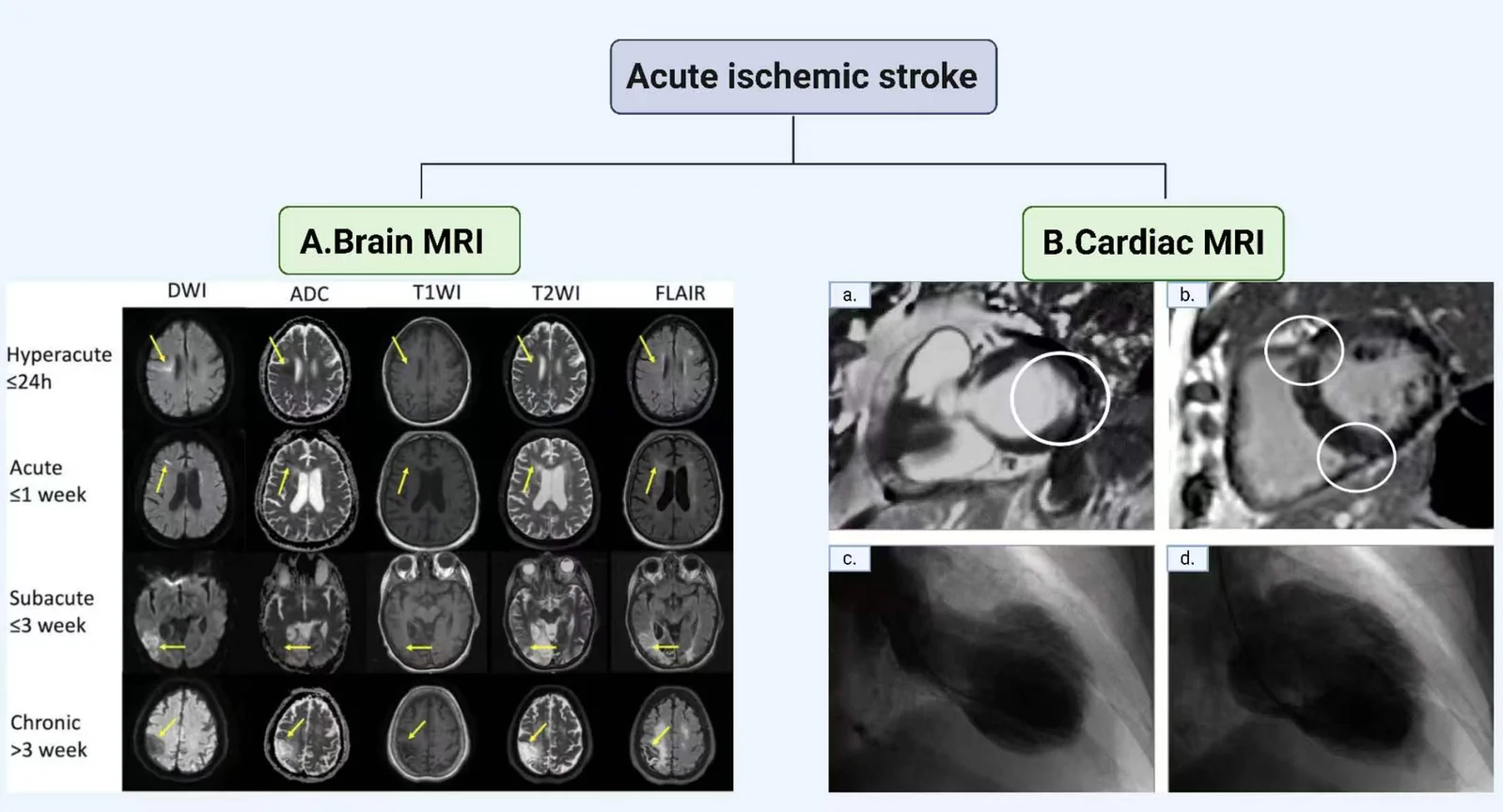
Multimodal MRI characteristics of stroke-heart syndrome following acute ischemic stroke. **(A)** Brain MRI findings at different stages of ischemic stroke—hyperacute (≤24 h), acute (≤1 week), subacute (≤3 weeks), and chronic (>3 weeks)—shown on diffusion-weighted imaging (DWI), apparent diffusion coefficient (ADC), T1-weighted imaging (T1WI), T2-weighted imaging (T2WI), and fluid-attenuated inversion recovery (FLAIR) sequences. Yellow arrows indicate the ischemic lesions. **(B)** Representative cardiac MRI findings after acute stroke: (a) subendocardial late gadolinium enhancement (LGE; white circle) in the anterolateral basal segment of the left ventricle, consistent with focal fibrosis with an ischemic pattern; (b) focal LGE at the right ventricular insertion points (white circles), consistent with a nonischemic pattern; and (c, d) apical wall-motion abnormalities associated with Takotsubo syndrome during systole and diastole, respectively. Panels in **(A)** and **(B)** were reproduced from Xiao et al. (55) and Stengl et al. (56), respectively. Panel **(A)** is reproduced with permission from the Radiological Society of North America, Inc. (CCC License ID: 1793050-1). Panel **(B)** is reproduced with permission from BMJ Publishing Group Ltd. (Copyright © BMJ Publishing Group Ltd.; CCC License ID: 1792836-1).

### 4.2 cognitive impairment related to heart failure

Heart failure(HF) is a common clinical disease characterized by a decrease in the blood supply of the heart due to anatomical or functional problems with the heart. In China, studies on the epidemiology of HF have revealed an increase in the prevalence rate among people over 35, from 0.9% in 2000 to 1.3% in 2019 (57). Ischemic cardiomyopathy and hypertension are considered major causes of HF and are associated with cognitive function. Decreased systolic function of the heart and blood redistribution are symptoms of HF, which is frequently a subsequent condition to myocardial infarction (58, 59). The fundamental etiology of brain functional injury is thought to be the decrease in cerebral perfusion pressure brought on by decreased cardiac output and inflammation worsened by oxidative stress (60, 61). Although anatomical abnormalities in the brain were linked to biomarkers of myocardial injury and cardiac failure, only older people with poorer cognitive reserves displayed cognitive deficits (62, 63). Conventional MRI is only applicable to lesions with significant brain structure and cannot fully identify the pathological mechanism. White matter hyperintensity (WMH) was connected to sorrow and anxiety, and atrophy of the medial temporal lobe was connected to cognitive problems such as memory loss and executive dysfunction in HF patients. Reduced hippocampal volume is present in HF patients, which may contribute to depression and short-term memory loss (64). fMRI further evaluates neural activity and functional connectivity by measuring blood oxygenation and cerebral blood flow changes, revealing reduced activation in autonomic and cognitive regulatory regions such as the insula, hypothalamus, anterior cingulate cortex, cerebellum, and hippocampus (58), which may contribute to impaired attention, emotional regulation, and working memory in HF. Structural MRI (sMRI), particularly when combined with advanced analytical techniques such as voxel-based morphometry and surface-based morphometry, enables quantitative assessment of gray matter density, cortical thickness, and white matter integrity, demonstrating widespread gray matter loss in the medial temporal lobe, hippocampus, prefrontal cortex, and precuneus that correlates with cognitive decline and HF severity (65, 66). In addition, WMH detected on T2-weighted or FLAIR imaging is closely associated with impaired information processing speed and executive dysfunction (67, 68). Cerebral perfusion imaging, including ASL, provides a noninvasive assessment of cerebral blood flow and microcirculation, showing reduced perfusion in the frontal cortex, hippocampus, thalamus, parietal lobe, and cerebellum of HF patients (58), suggesting that chronic cerebral hypoperfusion may contribute to neuronal injury and cognitive deterioration. Overall, the integration of conventional MRI, fMRI, sMRI, and perfusion imaging offers a comprehensive framework for understanding the heart-brain interaction in HF-associated cognitive impairment and may improve early diagnosis, mechanistic exploration, and therapeutic monitoring (15).

Representative multimodal MRI findings of heart failure-associated brain injury and cognitive impairment are illustrated in Figure 4. In patients with heart failure, reduced cardiac output and chronic hemodynamic impairment may result in sustained cerebral hypoperfusion and progressive neurovascular dysfunction. Functional MRI and advanced imaging analyses reveal alterations in large-scale cognitive and autonomic regulatory networks. Compared with controls, patients with HF showed significant ADC changes in multiple brain regions, primarily in the frontal, parietal, occipital, and temporal lobes (69). Collectively, these multimodal MRI signatures provide imaging evidence of chronic heart-brain coupling and highlight the impact of cardiovascular dysfunction on cerebral structure, perfusion, and functional network organization.

**Figure 4:**
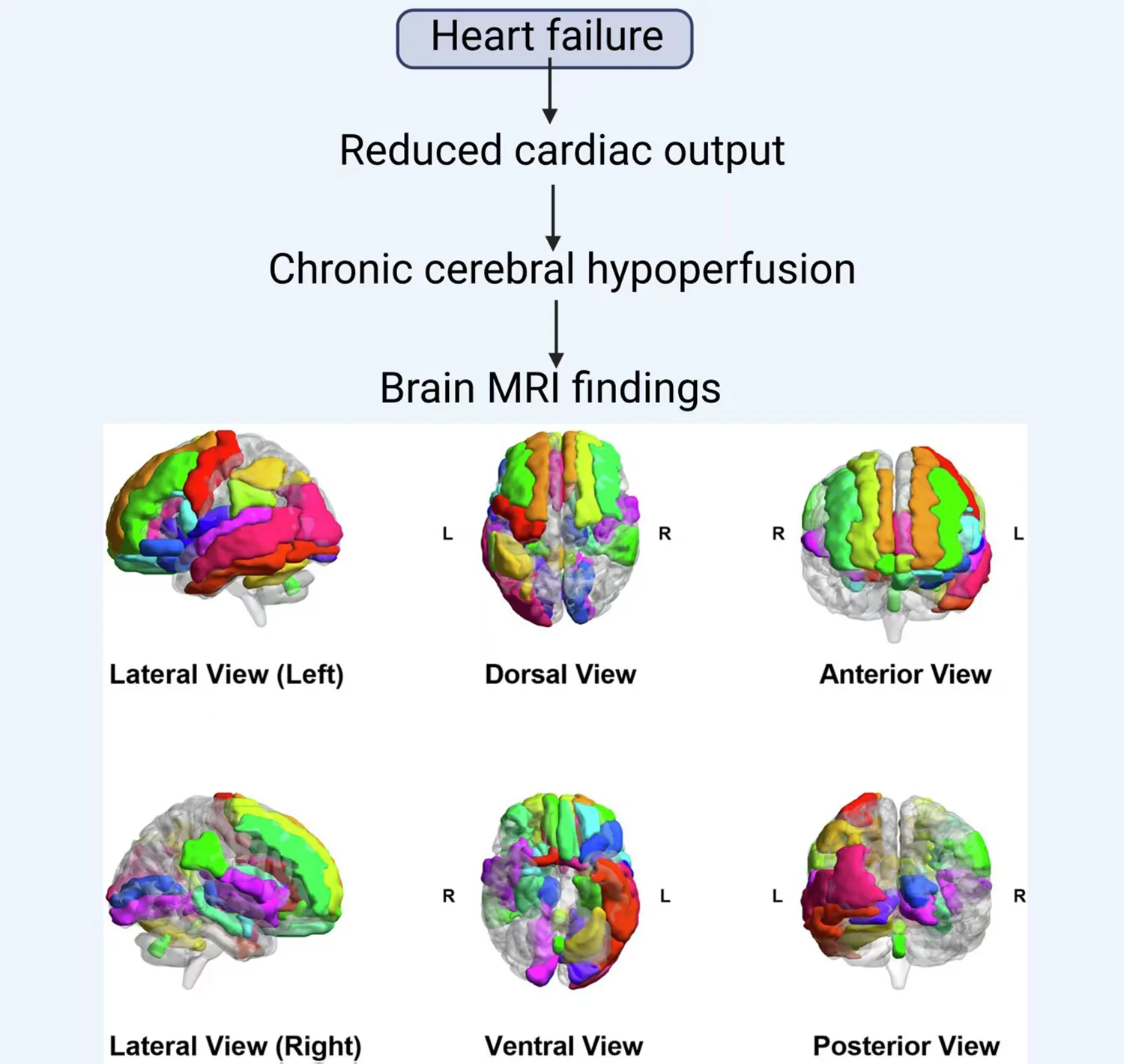
Structural brain alterations associated with heart failure-related cognitive impairment revealed by MRI. The flowchart illustrates the proposed pathway from heart failure to reduced cardiac output, chronic cerebral hypoperfusion, and brain MRI abnormalities. The six three-dimensional brain views show regions with increased apparent diffusion coefficient (ADC) values in patients with heart failure, including areas in the frontal, temporal, parietal, and occipital lobes, insula, cingulate cortex, hippocampus, amygdala, and cerebellum. L, left; R, right. Reproduced from Zheng et al. (69) with permission from Elsevier (License Number: 6351390773988).

### 4.3 cardiovascular abnormalities associated with neurodegenerative diseases

Neurodegenerative diseases such as AD are characterized by progressive neuronal loss, disruption of large-scale neural networks, and chronic autonomic dysfunction. Increasing evidence suggests that AD is frequently accompanied by cardiovascular abnormalities, including impaired heart rate variability, autonomic imbalance, arrhythmias, and subclinical myocardial dysfunction, indicating chronic remodeling along the heart-brain axis. Research by Aili Toyli and colleagues has increasingly illuminated a complex, bidirectional relationship between cardiovascular disease and AD (70). Unlike acute brain injury-related cardiac dysfunction, neurodegeneration-associated heart-brain remodeling develops gradually and is characterized by chronic neurovascular dysfunction, persistent autonomic dysregulation, cerebral hypoperfusion, and progressive network reorganization. Multimodal MRI provides important insights into the structural, functional, and neurovascular mechanisms underlying these chronic interactions. Structural MRI studies consistently demonstrate hippocampal and medial temporal lobe atrophy in AD (71, 72), often accompanied by increased white matter hyperintensities, suggesting concomitant cerebral small vessel disease and chronic ischemic injury (73). ASL further reveals reduced cerebral blood flow in temporoparietal and posterior cingulate regions, closely associated with cognitive decline. DTI and NODDI show reduced white matter integrity and axonal density, particularly within hippocampal-prefrontal and limbic pathways. fMRI studies further demonstrate disrupted connectivity within the DMN, salience network, and memory-related circuits, with DMN dysfunction considered a hallmark imaging feature of AD. Importantly, DTI and fMRI can detect structural and functional abnormalities before overt clinical symptoms, supporting their value in early diagnosis (74). In addition to cerebral abnormalities, patients with AD frequently exhibit autonomic and cardiovascular dysfunction, including reduced heart rate variability, impaired baroreflex sensitivity, orthostatic hypotension, and altered sympathetic-parasympathetic balance. Chronic autonomic dysregulation may further impair cerebral perfusion and vascular homeostasis, thereby forming a vicious cycle between neurodegeneration and cardiovascular dysfunction. CMR studies also suggest the presence of subclinical myocardial remodeling and impaired cardiac function in some patients with AD (75). Recent advances in multimodal MRI and artificial intelligence have further improved the diagnostic and predictive capability of neuroimaging in AD. The integration of structural MRI, ASL, DTI, rs-fMRI, MRS, and machine learning enables multidimensional assessment of brain atrophy, perfusion deficits, white matter degeneration, metabolic abnormalities, and network dysfunction, providing promising tools for early diagnosis, longitudinal monitoring, and precision intervention in AD-related heart-brain axis remodeling (76, 77).

In neurodegenerative disorders, chronic neuronal loss and progressive disruption of large-scale brain networks may gradually impair autonomic regulation and contribute to cardiovascular dysfunction. Alzheimer's disease provides a representative model of this chronic brain-to-heart interaction, in which structural degeneration, perfusion abnormalities, and functional network disruption coexist with autonomic and cardiovascular alterations. Multimodal MRI enables comprehensive characterization of these cross-organ changes, revealing the complex relationship between neurodegeneration and cardiovascular remodeling (Figure 5) (78–81).

**Figure 5:**
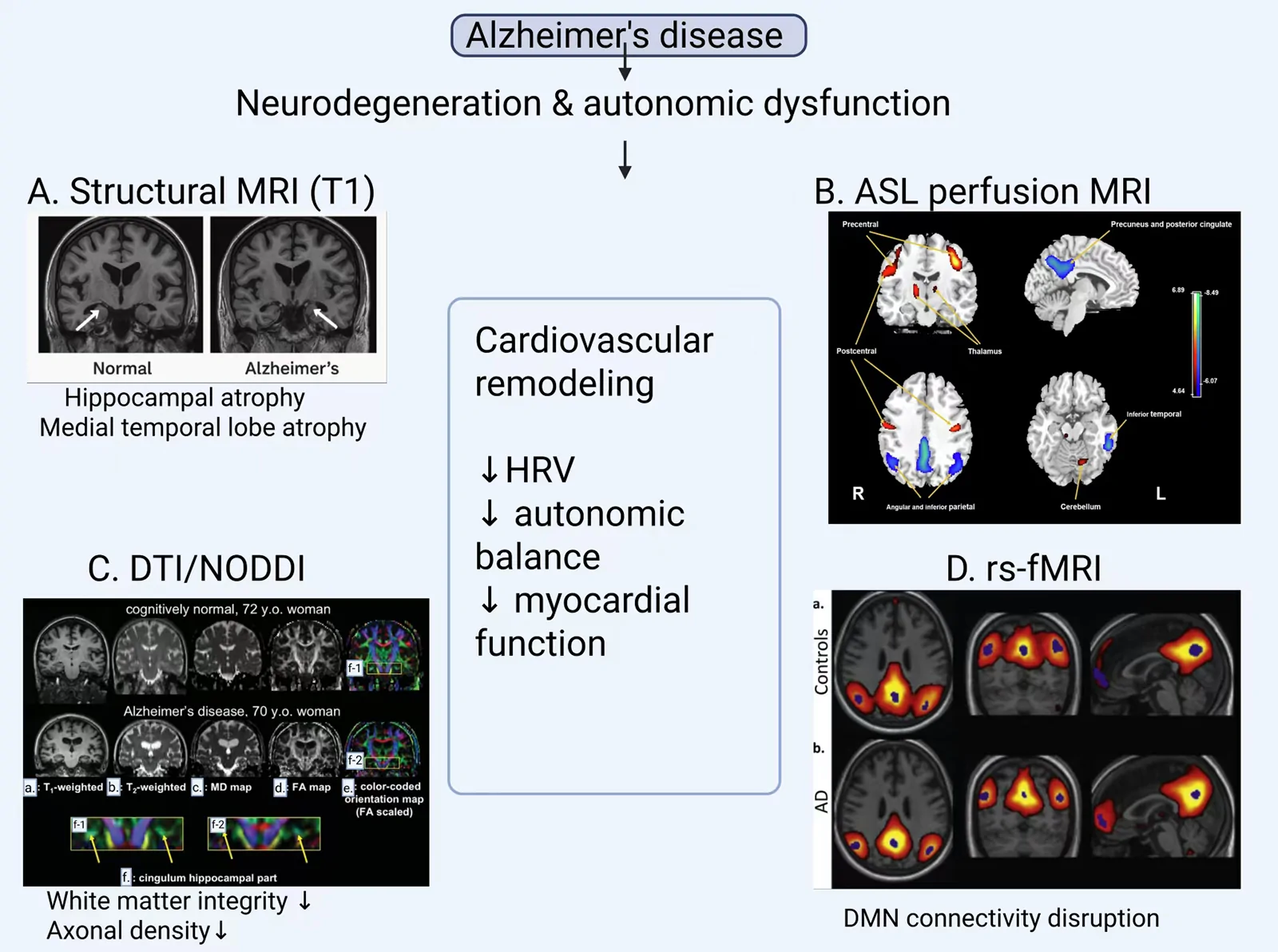
Multimodal MRI signatures of Alzheimer's disease-associated heart -brain axis remodeling. **(A)** Comparison of hippocampal volume and cortical thickness on structural MRI in a cognitively normal individual and a patient with Alzheimer's disease. **(B)** Regional cerebral blood-flow changes measured with arterial spin labeling (ASL) perfusion MRI in patients with Alzheimer's disease compared with cognitively unimpaired controls; cool and warm colours indicate relatively decreased and increased perfusion, respectively. **(C)** Comparison of conventional T1- and T2-weighted images and diffusion tensor imaging (DTI)-derived mean diffusivity (MD), fractional anisotropy (FA), and colour-coded orientation maps in a cognitively normal individual and a patient with Alzheimer's disease. **(D)** Z-transformed default mode network (DMN) components obtained by group independent component analysis in controls and patients with Alzheimer's disease. AD, Alzheimer's disease; ASL, arterial spin labeling; CBF, cerebral blood flow; DTI, diffusion tensor imaging; FA, fractional anisotropy; MD, mean diffusivity; DMN, default mode network. Panels **(A–D)** were reproduced from Liu et al. (78), Che et al. (79), Oishi et al. (80), and Mohtasib et al. (81), respectively. Panel **(A)** is reproduced under the Creative Commons Attribution -NonCommercial -NoDerivatives 4.0 International License (CC BY-NC-ND 4.0). Panel **(B)** is reproduced under the CC BY-NC-ND 4.0 license. Panel **(C)** is reproduced with permission from SAGE Publications (License Number: 6351391265787). Panel **(D)** is reproduced under the Creative Commons Attribution (CC BY) license.

## 5 Current challenges and future perspectives

Although multimodal magnetic resonance imaging has advanced heart-brain axis research from single-organ assessment toward cross-organ systemic characterization, the field remains largely transitional between correlation-based observation, mechanistic inference, and clinical translation. Current limitations include insufficient causal inference, the predominance of cross-sectional studies, limited multimodal integration, and incomplete characterization of dynamic heart-brain coupling. In addition, the lack of standardized acquisition protocols and unified cross-scale analytical frameworks across structural, functional, perfusion, metabolic, and ultra-high-field MRI restricts reproducibility and cross-center generalizability (82).

Artificial intelligence is increasingly reshaping heart-brain axis imaging research from experience-driven interpretation toward data-driven systems modeling. Radiomics and deep learning enable quantitative characterization of complex imaging phenotypes and improve disease classification and risk prediction. Emerging graph neural networks and Transformer-based models further facilitate cross-organ network analysis by capturing nonlinear interactions among brain networks, cardiac remodeling, and autonomic regulation. However, current AI applications remain limited by insufficient interpretability, inadequate external validation, and weak integration with biological mechanisms, which constrain clinical translation (83).

Molecular imaging represents another promising direction for future heart-brain axis research (84). Although MRI provides excellent structural, functional, and metabolic characterization, molecular imaging modalities such as PET offer unique advantages in detecting cellular metabolism, neuroinflammation, protein aggregation, and receptor-specific alterations (85, 86). The integration of PET with MRI may provide complementary molecular, anatomical, and functional information, enabling a more comprehensive understanding of heart-brain interactions (87). Future development of hybrid PET/MRI platforms, combined with advanced MRI techniques and artificial intelligence-based image fusion methods, may facilitate characterization of disease processes from molecular abnormalities to organ-level remodeling (88).

Large-scale cohorts such as the UK Biobank further support integrative analyses combining multimodal MRI with genetic, physiological, and clinical data (89). The incorporation of genome-wide association studies and Mendelian randomization approaches may strengthen causal inference and improve mechanistic understanding of heart-brain interactions (90, 91). Although observational MRI studies have demonstrated significant associations between cardiac remodeling and cerebral structural alterations, causal inference remains limited. Emerging Mendelian randomization approaches provide a promising framework for investigating directional heart-brain interactions through the integration of genome-wide association study data derived from cardiac and brain MRI phenotypes. Future bidirectional MR analyses may help determine whether genetically predicted cardiac traits, such as left ventricular mass, myocardial fibrosis, and ventricular dysfunction, causally contribute to hippocampal atrophy, cortical thinning, and white matter injury. Such studies may further clarify the directionality of heart-brain interactions and advance mechanistic understanding of the heart-brain axis (92).

Although multimodal MRI provides a comprehensive framework for characterizing heart-brain interactions, its translation into routine clinical practice remains challenging. Advanced approaches, including quantitative MRI, multinuclear MRI, metabolic imaging, and integrated heart-brain imaging protocols, are currently available mainly in specialized centers due to limited accessibility, heterogeneous acquisition strategies, and the lack of standardized protocols. Beyond technical availability, clinical implementation also requires streamlined scanning workflows, effective multidisciplinary collaboration, and automated analysis platforms to enhance operational efficiency and clinical feasibility. Moreover, the substantial costs associated with advanced imaging technologies, specialized hardware, and computational infrastructure emphasize the importance of identifying high-risk populations who are most likely to benefit from these approaches. Large-scale prospective and multicenter studies are therefore needed to validate clinical utility, determine prognostic value, and assess cost-effectiveness before widespread adoption in routine care and reimbursement frameworks. Overcoming these challenges through technological innovation, protocol harmonization, and collaborative research efforts will be critical for translating heart-brain axis imaging from an emerging research tool into a practical approach for personalized risk stratification and precision medicine.

Future research should therefore emphasize longitudinal cohort designs, standardized multimodal imaging protocols, dynamic temporal modeling (93), and interpretable deep multimodal fusion frameworks (94). With continued advances in integrated imaging, ultra-high-field MRI, and AI-assisted systems modeling, heart-brain axis research is expected to progress from static correlation analysis toward mechanism-driven precision medicine frameworks for early detection and personalized intervention in heart-brain comorbidities.

## 6 Conclusion

This review highlights that the heart-brain axis is not merely a conceptual framework for qualitative description but a complex cross-organ biological system that can be quantitatively characterized through multimodal magnetic resonance imaging. Collectively, these findings indicate that cardiac and cerebral injuries are more likely to reflect a coordinated systemic pathological process than independent organ-specific events. Nevertheless, current evidence remains largely observational and cross-sectional, limiting the ability to establish causal directionality and temporal dynamics within heart-brain interactions. Future studies integrating longitudinal multimodal MRI, genetic epidemiology approaches such as Mendelian randomization, and advanced causal inference frameworks may help clarify whether cardiac abnormalities precede cerebral degeneration, whether brain dysfunction drives cardiovascular remodeling, or whether both arise from shared pathogenic mechanisms. In addition, the incorporation of dynamic network modeling, graph-based artificial intelligence algorithms, and time-resolved imaging biomarkers may enable more precise characterization of the spatiotemporal evolution of heart-brain coupling across different stages of disease. With continued advances in multimodal MRI data integration, longitudinal modeling, and AI-assisted causal analytics, heart-brain axis research is expected to progress beyond the identification of associations toward the delineation of causal pathways and dynamic interaction patterns between the heart and the brain. Such efforts may uncover critical driver nodes, disease initiation sites, and therapeutic windows of opportunity, ultimately facilitating earlier detection, more accurate risk stratification, and precision intervention strategies for heart-brain comorbidities.
